# Physiological Response of the Hard Coral *Pocillopora verrucosa* from Lombok, Indonesia, to Two Common Pollutants in Combination with High Temperature

**DOI:** 10.1371/journal.pone.0142744

**Published:** 2015-11-10

**Authors:** Pia Kegler, Gunilla Baum, Lisa F. Indriana, Christian Wild, Andreas Kunzmann

**Affiliations:** 1 Department of Ecology, Leibniz Center for Tropical Marine Ecology, Bremen, Germany; 2 Mataram Marine Bio Industry Technical Implementation Unit, Research Center for Oceanography, Indonesian Institute of Sciences, Pemenang, Indonesia; 3 Faculty of Biology and Chemistry, University of Bremen, Bremen, Germany; King Abdullah University of Science and Technology, SAUDI ARABIA

## Abstract

Knowledge on interactive effects of global (e.g. ocean warming) and local stressors (e.g. pollution) is needed to develop appropriate management strategies for coral reefs. Surfactants and diesel are common coastal pollutants, but knowledge of their effects on hard corals as key reef ecosystem engineers is scarce. This study thus investigated the physiological reaction of *Pocillopora verrucosa* from Lombok, Indonesia, to exposure with a) the water-soluble fraction of diesel (determined by total polycyclic aromatic hydrocarbons (PAH); 0.69 ± 0.14 mg L-1), b) the surfactant linear alkylbenzene sulfonate (LAS; 0.95 ± 0.02 mg L-1) and c) combinations of each pollutant with high temperature (+3°C). To determine effects on metabolism, respiration, photosynthetic efficiency and coral tissue health were measured. Findings revealed no significant effects of diesel, while LAS resulted in severe coral tissue losses (16–95% after 84 h). High temperature led to an increase in photosynthetic yield of corals after 48 h compared to the control treatment, but no difference was detected thereafter. In combination, diesel and high temperature significantly increased coral dark respiration, whereas LAS and high temperature caused higher tissue losses (81–100% after 84 h) and indicated a severe decline in maximum quantum yield. These results confirm the hypothesized combined effects of high temperature with either of the two investigated pollutants. Our study demonstrates the importance of reducing import of these pollutants in coastal areas in future adaptive reef management, particularly in the context of ocean warming.

## Introduction

With growing human populations, the anthropogenic influence on coastal ecosystems is increasing. Halpern et al. (2008) found that no marine areas are unaffected by anthropogenic influences and 41% are even strongly affected [1]. About 275 million people live in close vicinity to coral reefs and most of them depend on their ecosystem services for their livelihoods [2]. One third of the world’s coral reefs are located in the Coral Triangle in the Indonesian/Philippines Archipelago [2], where hard coral cover declined significantly within the past decades due to a multitude of global and local stressors (i.e. factors that are diverging from the natural conditions) [3]. About 85% of all reefs within the coral Triangle are threatened by local stressors, up to 90% in combination with global stressors [2]. Global stress is generated by climate change, which is usually accompanied by local anthropogenic drivers, such as overfishing, pollution, sedimentation and eutrophication, which in combination result in enhanced vulnerability of the ecosystem [4–6]. Global sea surface temperatures are estimated to increase up to 4.8°C within this century [7]. Low variances in surface temperature in tropical regions such as Southeast Asia, where organisms live already close to their upper thermal limits, leave organisms there more susceptible to climate change [8,9]. Bleaching of corals due to a breakdown of the symbiosis between corals and their symbiotic algae is often times strongly related to high sea surface temperatures, as shown in field and laboratory studies [10–13]. The majority of studies concerning stressors on coral reefs have focused on ocean acidification and global warming associated coral bleaching. Several studies have investigated combined effects where stressors can have additive, synergistic or antagonistic effects, and it is important that we understand these in order to develop appropriate management strategies for coral reefs [14,15]. Synergistic effects have been found for example between temperature and light stress on photosynthesis in corals [16], while increased CO_2_ levels and temperature had antagonistic effects [17].

One important pollutant in the ocean is diesel, used to fuel machines and ships all over the world. Although diesel is not the only source of oil pollution (there are many others, both from anthropogenic, as well as natural sources, see [18]), it is a very important one. In 2012 each day over 3.5 billion liters of motor diesel were consumed all over the world and while an effort is made to reduce this number, in growing countries like in the coral triangle region, there was a steady increase in diesel consumption over the past decade (e.g. in Indonesia from 40 million L d^-1^ to 84 million L d^-1^) [19]. Diesel is introduced to the environment via oil spills from ships and harbors, from discharge of routine tanker operations and from municipal and urban runoff [20,21]. Among the water-soluble constituents in diesel, polycyclic aromatic hydrocarbons (PAHs) pose the highest threat to the environment [22,23]. Several studies have discovered toxic effects of PAH on aquatic organisms, mainly fish [21,24–26]. A range of physiological responses to oil pollution by corals, depending highly on the type of oil used, were found in previous studies, including growth impairments, mucus production and decreased reproduction (for an overview see [23]). But most of these studies were performed before 1990 investigating effects of large oil spills and due to the many different types of oils, concentrations and durations it is hard to compare the results [27].

Another group of frequently used chemicals that regularly end up in the ocean are surfactants, which are applied by households and industry in large quantities in detergents and soaps. In 2003 18.2 billion kg of surfactants were used all over the world [28]. Linear alkylbenzene sulfonate (LAS) is one of the most common surfactants in use [29], the consumption of LAS alone in 2003 was 2.9 billion kg [28]. Although to some extent surfactants are eliminated from water by biodegradation within a few hours up to several days, significant proportions of surfactants attach to suspended solids and remain in the environment [30]. This sorption of surfactants onto suspended solids depends on environmental factors, such as temperature, salinity or pH [30]. The important role of water temperature in combination with pollution is mainly due to enhanced reaction rates at higher temperatures, which leave organisms more sensitive to chemicals [14,31].

Indonesia, the country with the largest area of coral reefs within the coral triangle, has a large and growing number of human settlements clustered along the entire coastline in close vicinity to coral reefs, and in most cases no waste water treatment is occurring [2,20]. In areas without sufficient sewage- and waste water treatments, concentrations reaching 1.1 mg L^-1^ of LAS and 0.2 mg L^-1^ of PAH from diesel can be entering the reefs [29,31]. Thus, there is a continuous contamination of coastal waters with these two very commonly used pollutants, turning the local stressor into a regional threat.

While several studies have investigated responses to contaminants on the cellular level, it is important to understand the effects of stressors on physiological performance on the whole-organism level [32]. Metabolic rates are indicators of the overall energy budget of organisms and can indicate non-lethal stress responses [33]. Metabolism of the coral holobiont [34,35] includes host and symbiont respiration, as well as symbiont photosynthesis and metabolic energy is needed among other processes to transport calcium to the host skeleton [36, 37]. Kaniewska et al. (2012) showed that effects in coral physiology become apparent before any changes in calcification processes can be detected [38]. Thus, measurements of respiration in combination with photosynthesis are a common method in coral physiological research [9]. An increase in respiration can indicate acute stress, while a decrease can indicate either an acclimation or depression due to a stressor [39]. To investigate photosynthetic capacity, the quantum yield of linear electron transport is a useful tool to determine coral health and serves as a diagnostic tool for the analysis of pollutants [9,40]. Further, the ratio of photosynthesis to respiration (P:R) provides an estimate, whether a coral can live on the energy obtained from its zooxanthellae [41].

To our knowledge, there are no publications describing effects of the pollutants diesel and LAS each combined with high temperature on coral metabolism. This study investigates this potentially interactive effect on the physiology of a tropical reef coral *Pocillopora verrucosa* in acute exposure experiments. *P*. *verrucosa* is common in the study area and therefore a good representative of the scleractinian corals that are of vital importance for coral reefs. The main objective was to determine if and how the coral is affected by pollutants in isolation and combination with increased temperature. Special focus was put on whether there are combined effects between the pollutants and high temperature, because their simultaneous occurrence in the reef is likely. Oxygen consumption and photosynthetic activity were chosen as response parameters, to determine the response of the coral holobiont metabolism. The hypothesis is that the metabolism of *P*. *verrucosa* will be negatively affected by both pollutants and that there will be combined effects with temperature.

## Material and Methods

### Coral sampling and rearing

*Pocillopora verrucosa* fragments of approx. 5 cm height (average surface area ± SD: 112 cm^2^ ± 29 cm^2^) were sampled using Scuba diving from approx. 6–8 m water depth at two sites (S 08°20.259’, E 116°02.260’ and S 08°21.768’, E 116°01.897’) during 4 days in July 2013 on Gili Trawangan north of Lombok, Indonesia. Both sites were similar in reef habitat, environmental conditions and measured physical water parameters. The research permit for the study area was approved by the Indonesian ministry for research and technology (RISTEK, permit no. 176/SIP/FRP/SM/V/2013). Two fragments each from a total of 60 colonies were sampled from the two sites combined. All fragments were glued onto 5x5 cm ceramic tiles directly after sampling and brought to a rearing station located in the reef in front of the sampling island (S 08°20.750’, E 116°02.608’). They were left in the reef to recover from sampling for two weeks. Water parameters (salinity, temperature, pH and dissolved oxygen) at all sites were measured before and after each dive using a Eureka Manta 2 multiprobe (Eureka Water probes, Austin, USA) and water samples were taken for environmental LAS and PAH determination. After two weeks all healthy coral fragments (two fragments each from 42 colonies) were taken to a laboratory of the Indonesian Institute for Science (LIPI) at Pemenang, Lombok. Fragments were placed in a 600 L semi-flow-through outside tank, located in a larger pool to buffer temperature fluctuations during the day. Water flow through the tank was adjusted that the entire water volume was exchanged once a day with fresh water from the reef, supplied by a pump ca. 200 m away from the shore. Water circulation within the rearing tank was created by using two circulation pumps (Hydor korallia, Hydor Ind., Sacramento, USA). Light conditions at the sampling sites were measured using a LI-1400 data logger with a Li-192 underwater quantum light sensor (LI-COR Biosciences, Lincoln, USA). They ranged from 90 to 500 μmol quanta m^-2^ s^-1^ and averaged around 200 μmol quanta m^-2^ s^-1^ during midday, therefore were adjusted to these intensities in the rearing system by shading the tank from direct sunlight. Corals received additional heterotrophic feeding with designated coral food (Coral V Power by Preis Aquaristik, Bayerfeld, Germany) once a day. Temperature, salinity, pH and dissolved oxygen were monitored daily, always at the same time, using a WTW 340i Multiparameter system (WTW GmbH, Weilheim, Germany).

### Experimental protocol

Four main experiments were performed with four replicates for each treatment (see Table 1). Corals from the two sampling sites were randomly chosen for the different experiments. Treatments were control, increased water temperature, diesel and combination of diesel with increased temperature. For the control treatment the same protocol as for the other treatments was applied, but neither pollutant nor high temperature were applied. Two additional experiments were performed with the surfactant LAS (linear alkylbenzene sulfonate) alone and in combination with increased temperature. A reduced experimental protocol was used for these two treatments, where no respiration, but only photochemical yield was measured. Temperature was increased using Eheim Jäger 150 W aquarium heaters (Eheim GmbH & Co. KG, Deizisau, Germany). The control temperature was adjusted to 28°C, to resemble the temperature measured in the reef. Increased temperature was 31°C, three degrees above the control temperature. Diesel was bought at a local gas station and a water accumulated fraction (WAF) [25,42] was produced from 5 g diesel in 1 L of filtered seawater. This solution was stirred for 24 h, then left to settle for 20 min before the lower phase with the waterborne diesel constituents was retrieved. 490 mL of this 0.5% WAF were immediately administered to each tank at the start of the experiments, giving a final concentration of 0.0025%. LAS was purchased from a local supplier in Indonesia (PT. Findeco Jaya, www.findeco.com) and stored at 4°C. For each experiment 190 μL LAS were administered to each tank, resulting in a final concentration of 0.00019%.

**Table 1:** Treatments.

Treatment	Description
Control	control reef temperature ~28°C, without pollutant addition
High Temperature	+3°C, without pollutant addition
Diesel	490 mL of 0.5% water accumulated fraction (WAF) of diesel (final concentration 0.0025 %)
LAS	190 μL Linear alkylbenzene sulfonate (final concentration 0.00019 %)
Diesel and Temperature	490 mL of 0.5% WAF, +3°C
LAS and Temperature	190 μL, +3°C

List of all treatments administered to *Pocillopora verrucosa* during the study.

**Fig 1:**
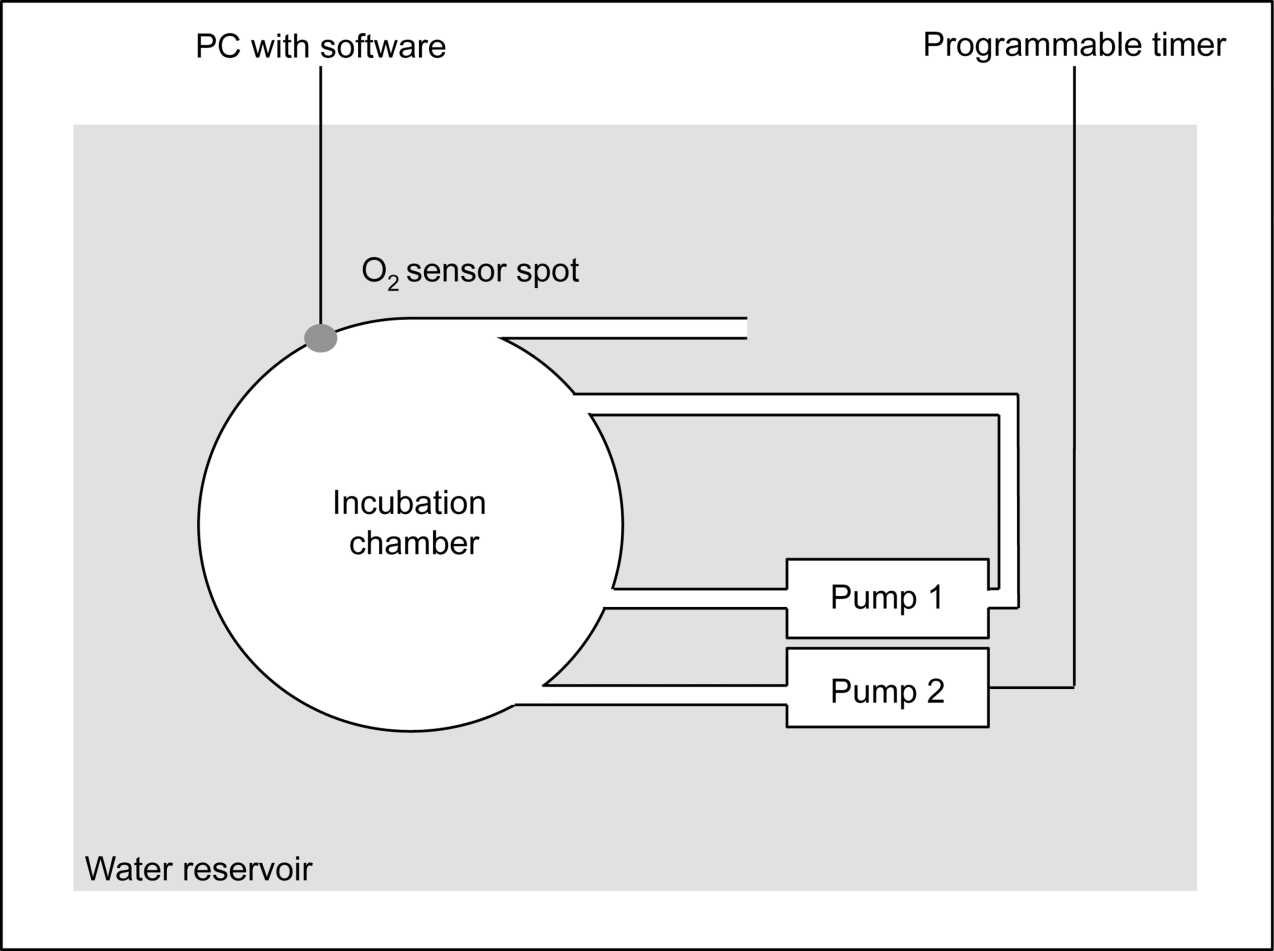
Schematic drawing of the experimental setting. Oxygen concentrations within the incubation chambers were continuously monitored using a sensor spot and optic fiber cable. A recirculation pump (pump 1) was used to ensure continuous water flow within the chamber, while pump 2 was turned on at programmed intervals to flush the chamber with oxygenated water from the surrounding water reservoir. Four incubation chambers were placed in the reservoir simultaneously, which served also as a temperature control.

All treatments were applied for a total of 84 h; first corals were subjected to 48 h of pre-treatment without measurements to increase the exposure time of the corals to the stressors. This was followed by 36 h of continuous oxygen measurement, during which also analysis of photosynthetic yield took place. During the pre-treatments the tanks were subjected to natural daylight adjusted to the same intensities as in the rearing tank, while the measurement tanks were artificially illuminated from 7:00 to 19:00, using a 2x20 W aquarium light (MW1-Y20X2 from Guangdong Zhenhua Electric Appliance Co. Ltd, Zhonhshan, China). The light intensities were 60 μmol quanta m^-2^ s^-1^ and a 12 h light: 12 h dark cycle was adjusted to simulate natural conditions. This difference in light intensity compared to the rearing and pre-treatment period occurred due to practical reasons of the laboratory set up, but were the same for all experiments. Pre-treatments always started in the evening and lasted for 48 h during which no heterotrophic feeding of the corals took place. Prior to the measurement phase all epiphytes and debris were removed from the coral fragments and the tiles. Then the fragments were moved to the respiration set-up (see Fig 1), which was prior to the start of measurements filled with filtered seawater and the same stressors as in the pre-treatment tanks. Physical water parameters were measured every day for each pre-treatment and measurement phase, using a WTW 340i Multiparameter system (WTW GmbH, Weilheim, Germany). Water samples from pre-treatment and measurement tanks were analyzed for pollutants as described below. After each experiment the entire experimental set-up was cleaned with 70% Ethanol and rinsed with distilled water to remove bacterial contamination. Surface area of the coral fragments was determined using the aluminum foil method described by Marsh (1970) [43]. In this method pieces of aluminum foil are fitted closely to the coral skeleton and the weight of these pieces is compared to a calibration curve with aluminum foil pieces of known size. After each experiment all fragments were photographed from two sides and the pictures analyzed using Image J software (v1.47, National Institutes of Health, Bethesda, USA) to determine the amount of tissue loss during experiments.

### Respirometry and PAM fluorometry

Measurements of oxygen fluxes took place in acrylic incubation chambers. To provide stronger oxygen fluxes and reduce the error due to individual variance, two fragments from one colony were measured in the same incubation chamber. Four of these chambers were situated within a 100 L tank, which served both as a reservoir used for flushing the incubation chambers with oxygenated water and to equalize the temperature between the replicates. Each chamber was connected to a pump ensuring water circulation within the chamber and to a flush-pump, supplying oxygenated water from the surrounding water bath at programmed time intervals using a custom build timer (see Fig 1 for details). 30 min measurement periods were followed by 3 min flush periods to allow for continuously high oxygen levels (>90% saturation) within the chambers. During the entire measurement phase oxygen content within the incubation chambers was recorded using optical oxygen sensor spots and a 4-channel Firesting oxygen meter with the associated software (Oxygen Logger v.3.12.4, Pyro Science GmbH, Aachen, Germany). The system was calibrated prior to each experiment. After the coral fragments were removed from the incubation chambers blank respiration in the system was measured for another 1.5 h to determine bacterial respiration at the end of the experiment. Oxygen fluxes from respiration and photosynthesis were calculated as described below. Photosynthetic capacity was determined by measuring the chlorophyll fluorescence of photosystem II (PS II), using a pulse-amplitude modulated fluorometer (DIVING-PAM, Heinz Walz GmbH, Effeltrich, Germany). Maximum quantum yield (F_v_/F_m_) [44] was measured in the beginning and end of the measurement phase in corals that were dark-adapted (1 h at the beginning of the experiment, approx. 10 h at the end of the experiment as it took place following the dark measurements). All fragments were measured three times with approx. 5 min between each measurement.

### LAS and PAH determination

Water samples from each experiment were taken at the beginning and end of each pre-treatment and measurement phase. To quantify the diesel WAF within the samples total polycyclic aromatic hydrocarbons (PAHs) were determined, which are among the major soluble toxic constituents in diesel and a practical measure for the toxicity of a PAH mixture [24]. Samples for PAH determination were filtered (0.7 μm filters, VWR International, Radnor, USA) and stored with 2-propanol (50 mL) before pre-concentrating them using solid-phase extraction (SPE) by passing it through a CHROMABOND C18 PAH cartridge (6 ml, 2000 ng; Macherey-Nagel GmbH & Co. KG, Düren, Germany) and elution of the PAHs with 5 mL dichlormethane from the cartridge. The dichloromethane was evaporated to 1 ml and 250 μl of dimethylformamid was added as keeper. For analysis of total EPA-PAH concentrations Ultra performance liquid chromatography (UPLC) was performed at the Institute for Chemistry and Biology of the Marine Environment (ICBM) in Oldenburg, Germany. A methodological quality control was performed, using a deuterated internal standard and the standard addition method. For LAS analysis triplicate samples of 50 mL were taken and stored at 4°C until further analysis. All LAS determination took place <24 h after sampling. Spectrophotometric analysis (SQ300 from Merck Millipore, Billerica, USA) was performed applying a modified version of the methylene blue assay for anionic surfactants (MBAS) standard method [45]. Prior to each LAS determination, a calibration curve was obtained using sodium-dodecyl sulfonate as a standard and filtered seawater from each experimental tank to ensure the same salinity.

### Data analysis

Oxygen fluxes were calculated using Microsoft Excel 2010. For each 30 min measurement period the decline in oxygen concentration within the incubation chamber was calculated and standardized to the consumption in 1 h. Fluxes were determined for each 12 h dark and light period (day and night) separately, taking averages from 18–25 measurement periods. All values were further standardized to surface area of the coral and values for bacterial respiration were accounted for by including the oxygen fluxes from the blanks, measured after the experiments. Gross photosynthetic rate was estimated as the difference between oxygen fluxes during dark and light periods (i.e. the dark respiration and net photosynthesis), assuming the same respiration rates during light and darkness (see discussion). To avoid any confounding effects due to handling stress after the pre-treatment, the first 12 h in each experiment were excluded from the analysis. Statistical analysis was performed in R (R v.3.0.2 using R Studio v.0.98.1056). All data were checked for normal distribution using the Shapiro Wilk test and for heterogeneity of variance with Levene’s test. Two-way ANOVA was carried out with diesel and temperature as fixed factors for dark and light periods separately to determine significant effects of the stressors and their interaction. To determine differences between the individual treatments, a post-hoc Tukey HSD test was applied (see S1 Table in the supporting information). In case of the photosynthetic yield in the LAS treatments, data were not normal distributed and multiple Wilcox rank sum tests were applied to detect differences between treatments.

## Results

### Water parameters

Physical water parameters measured within the rearing tank and during the experiments resembled those determined at the sampling sites (Table 2). LAS and PAH concentrations at the sampling sites were below detection limit, thus considered to reach zero. During the experiments there was no significant difference in the LAS or PAH concentrations between pre-treatment and measurement phase or between control and high temperature (p>0.05). The pollutant concentrations for both diesel (as measured by total PAH analysis) and LAS decreased significantly from the beginning to the end of each pre-treatment and measurement period. The values decreased from 0.69 ± 0.14 mg L^-1^ to 0.25 ± 0.05 mg L^-1^ and from 0.95 ± 0.02 mg L^-1^ to 0.87 ± 0.05 mg L^-1^ for PAH and LAS, respectively.

**Table 2:** Physical water parameters.

	Sampling stations	Coral keeping	Pre-treatments	Measurement phase
Salinity [PSU]	34.0 ± 0.2	33.7 ± 0.1	34.0 ± 0.2	34.1 ± 0.2
DO [% sat]	102.2 ± 3.0	102.8 ± 5.0	102.0 ± 1.1	96.3 ± 2.8
pH	8.1 ± 0.0	8.3 ± 0.0	8.3 ± 0.0	8.1 ± 0.1
Temp. control [°C]	28.3 ± 0.2	28.3 ± 0.5	28.9 ± 0.3	27.9 ± 0.1
Temp. high [°C]			31.1 ± 0.2	30.9 ± 0.1

Salinity, dissolved oxygen (DO), pH and temperature at the sampling stations (averages of both stations are shown), the rearing system and the experiments (average of the daily measured parameters at midday). Temperature for the experiments is given for the control and high treatments separately.

### Control

As a control corals were subjected to the same experimental protocol (including pre-treatment and measurement period) like the other treatments. Without any stressor present dark respiration rates were 0.0193 ± 0.0049 mgO_2_ h^-1^ cm^-2^ and net photosynthesis rates 0.0082 ± 0.0032 mgO_2_ h^-1^ cm^-2^ (see Table 3 for all results). The gross photosynthesis rate calculated from the difference between dark respiration and net photosynthesis was 0.0111 ± 0.0028 mgO_2_ h^-1^ cm^-2^, concluding in a P:R ratio of 0.58 ± 0.12. Maximum quantum yield did not differ between the start and end of the experiment, in both cases being 0.71 ± 0.02.

**Table 3:** Summary of *P*. *verrucosa* responses.

	Dark respiration [mgO_2_ h^-1^ cm^-2^]	Net photosynthesis [mgO_2_ h^-1^ cm^-2^]	Gross photosynthesis [mgO_2_ h^-1^ cm^-2^]	Maximum quantum yield 48 h [F_v_/F_m_]	Maximum quantum yield 84 h [F_v_/F_m_]	Tissue loss after 84 h [% loss]
Control	0.019 ± 0.005	0.008 ± 0.003	0.011 ± 0.003	0.71 ± 0.02	0.71 ± 0.02	-
High temperature	0.012 ± 0.003	0.003 ± 0.001	0.009 ± 0.003	0.74 ± 0.01	0.72 ± 0.01	-
Diesel	0.015 ± 0.001	0.006 ± 0.003	0.001 ± 0.003	0.71 ± 0.02	0.71 ± 0.02	-
LAS	-	-	-	0.73 ± 0.01		52.5 ± 30.15
Diesel + high temperature	0.023 ± 0.003	0.008 ± 0.002	0.014 ± 0.005	0.74 ± 0.01	0.71 ± 0.01	-
LAS + high temperature	-	-	-	0.63 ± 0.13		92.25 ± 7.26

Physiological responses of the coral holobiont for all treatments. Given are holobiont dark respiration, net and gross photosynthesis and maximum quantum yield measured after 48 h and 84 h. Maximum quantum yield after 84h and respiration in treatments containing LAS were not measured due to the separation of coral host and symbiotic algae by tissue loss. (see LAS results and discussion section). Tissue loss as seen in treatments containing LAS is given as determined at the end of the experiment.

### Diesel

**Fig 2:**
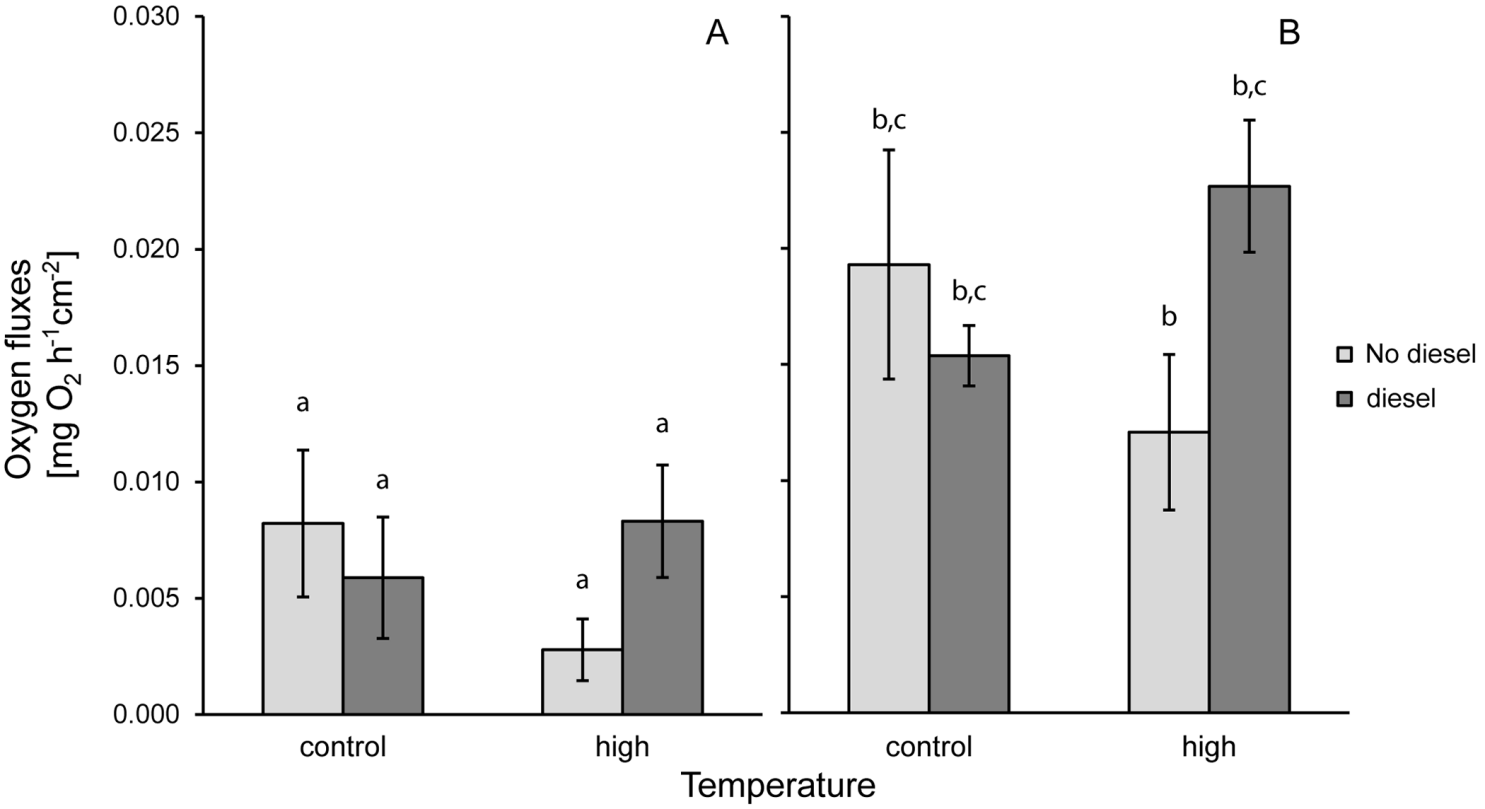
Physiological responses to diesel and high temperature. Fluxes of oxygen in diesel and high temperature treatments with *Pocillopora verrucosa* for the light and dark period. Net photosynthesis (A) and dark respiration (B) are presented. Given are averages for each treatment (n = 4) with standard deviations. Different letters indicate significant difference as found in Tukey HSD post hoc test.

**Fig 3:**
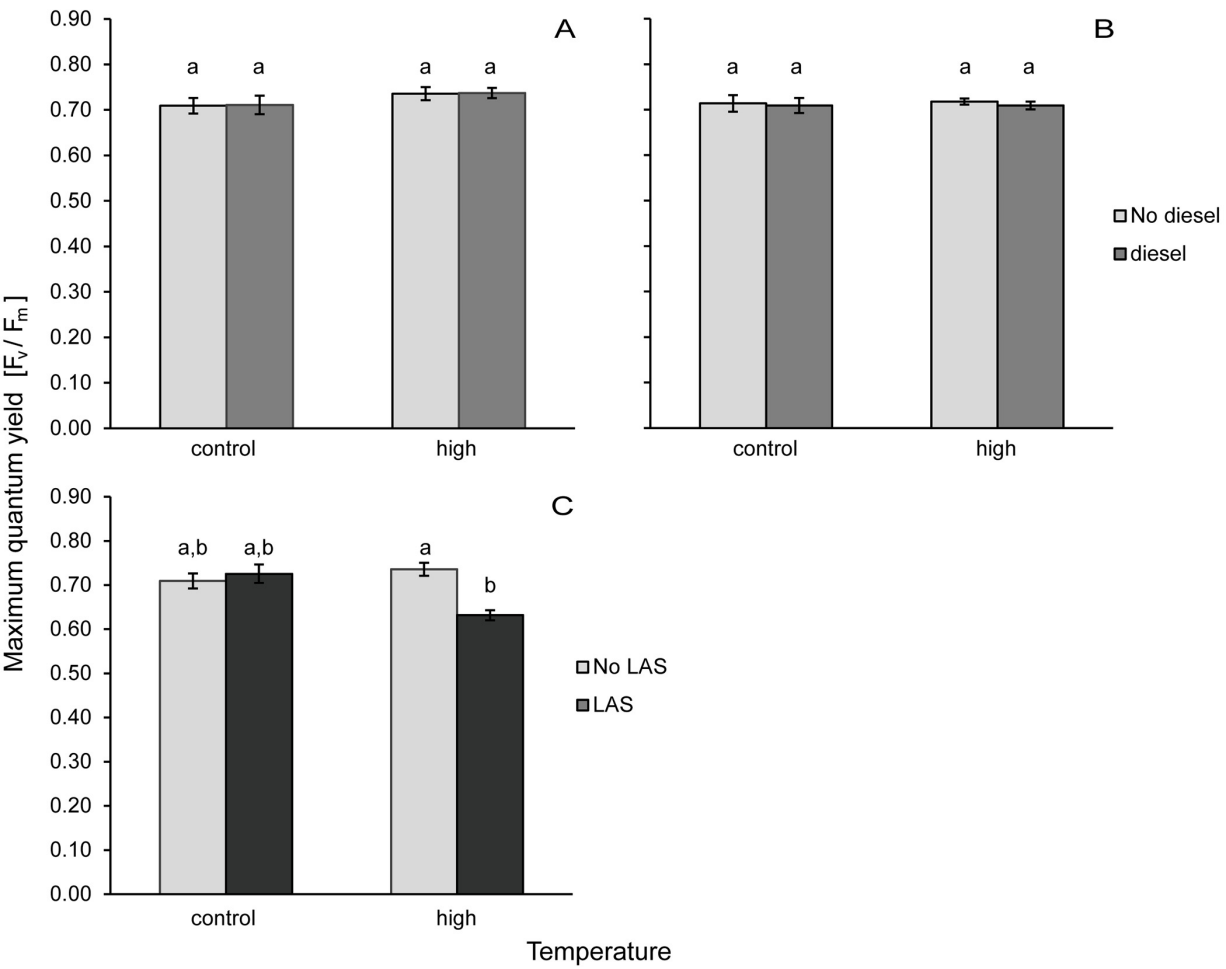
Maximum quantum yield. F_v_/F_m_ of *Pocillopora verrucosa* subjected to pollutants and high temperature. WAF of diesel (A+B) and linear alkylbenzene sulfonate, LAS (C) were administered either individually or in combination with high temperature. Measurements took place after 48 h and 84 h (A+C and B, respectively). Due to high tissue loss in treatments containing LAS no yield was measured in these treatments after 84h. Given are averages for each treatment (n = 4) with standard deviation. Different letters indicate significant differences as determined by Wilcox rank sum tests.

When corals were exposed to the water accumulated fraction of diesel, the dark respiration rates and net photosynthesis were 0.0154 ± 0.0013 mgO_2_ h^-1^ cm^-2^ and 0.0059 ± 0.0026 mgO_2_ h^-1^ cm^-2^, respectively (Fig 2). Gross photosynthesis was 0.0095 ± 0.0030 mgO_2_ h^-1^ cm^-2^ with a P:R ratio of 0.61 ± 0.17. The maximum quantum yield gave exactly the same results as in the control treatment with 0.71 ± 0.02 (Fig 3). No significant effects of diesel exposure were detected on either of these parameters (see Table 4 for ANOVA results).

**Table 4:** ANOVA results for diesel and temperature experiments.

	Df	(SS)	(MS)	F value	Pr (>F)
**F**_**v**_ **/F**_**m**_ **(Beginning of measurement period)**					
Temperature	1	3.03E-03	3.03E-03	8.963	0.011 *
Pollutant	1	0.00E+00	0.00E+00	0.000	1.000
Temperature:Pollutant	1	2.50E-05	2.50E-05	0.074	0.790
**F**_**v**_ **/F**_**m**_ **(End of measurement period)**							
Temperature	1	2.50E-05	2.50E-05	0.090	0.770
Pollutant	1	1.00E-04	1.00E-04	0.358	0.561
Temperature:Pollutant	1	1.00E-04	1.00E-04	0.358	0.561
**Net photosynthesis**					
Temperature	1	8.93E-06	8.93E-06	1.100	0.315
Pollutant	1	1.02E-05	1.02E-05	1.252	0.285
Temperature:Pollutant	1	6.17E-05	6.17E-05	7.601	0.017 *
**Dark respiration**					
Temperature	1	1.00E-08	1.00E-08	0.001	0.982
Pollutant	1	4.46E-05	4.46E-05	2.947	0.112
Temperature:Pollutant	1	2.11E-04	2.11E-04	13.939	0.003 *

Two-way analysis of variance (ANOVA) for maximum quantum yield (F_v_/F_m_) and respiration values. Effects of temperature (control or high) and pollutant (no pollutant or with diesel) were analyzed in isolation and in combination with each other. Significant values (p<0.05) are indicated by an asterisk.

### Temperature

Similar to diesel, temperature on its own had no effect on the oxygen fluxes or P:R ratio of the coral. Dark respiration was 0.0121 ± 0.0034 mgO_2_ h^-1^ cm^-2^, net photosynthesis was 0.0028 ± 0.0013 mgO_2_ h^-1^ cm^-2^ (Fig 2), concluding in a gross photosynthesis of 0.0093 ± 0.0032 mgO_2_ h^-1^ cm^-2^ and a P:R ratio of 0.76 ± 0.11. At the start of the measurement phase, after corals were exposed to high temperature for 48 h, maximum quantum yield was significantly increased compared to all other treatments (p = 0.0112, confirmed by Tukey HSD). At this time the yield was 0.74 ± 0.01, but subsequently decreased to control levels of 0.72 ± 0.01 within 24 h until the end of the experiment (Fig 3).

### LAS

**Fig 4:**
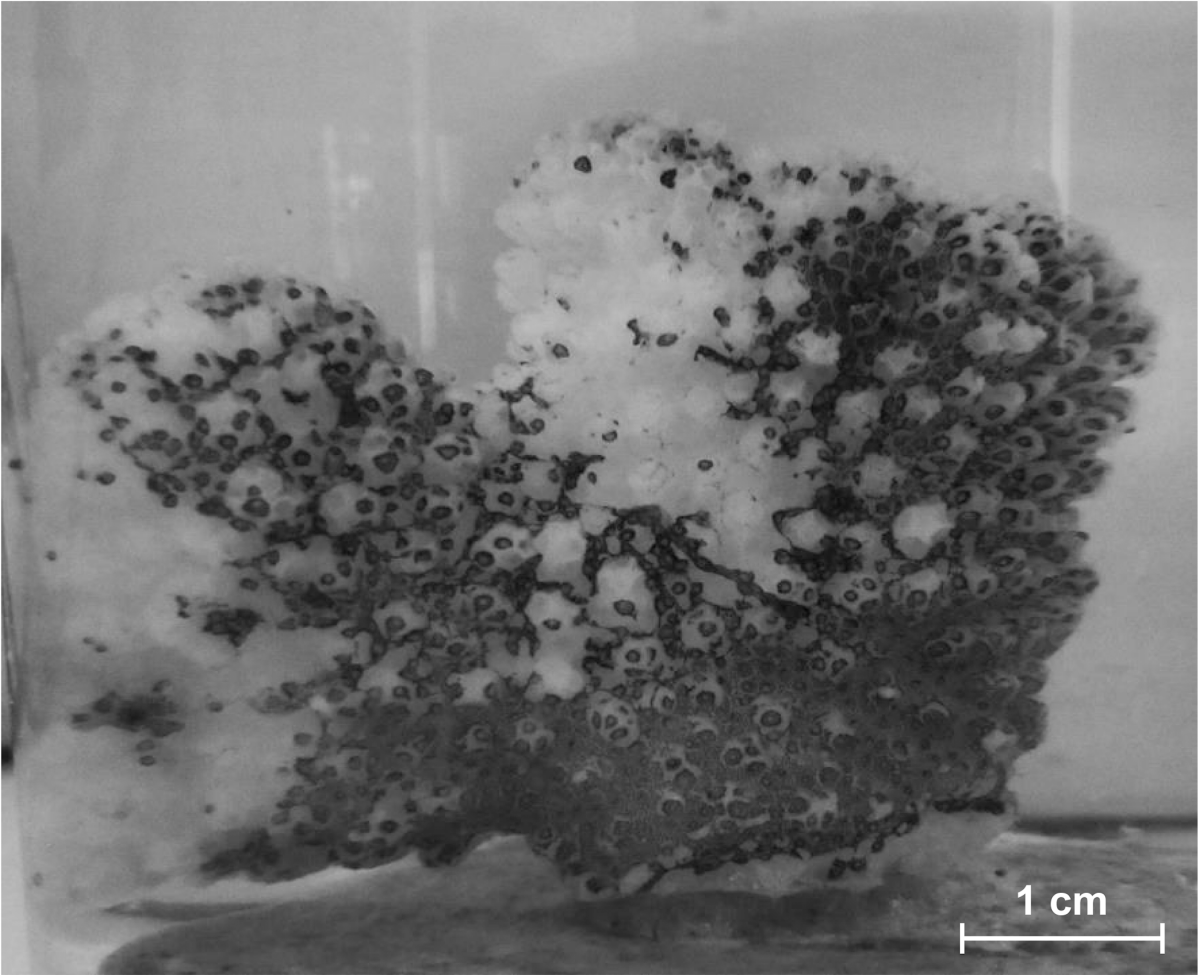
Tissue loss due to LAS. *Pocillopora verrucosa*, subjected to LAS treatment, showing severe tissue loss after 84 h exposure.

Exposure (>24 h) to LAS caused tissue ablations in the coral fragments (see Fig 4). This ranged from 16% tissue loss in some fragments up to 95% in others at the end of the experiment (84 h). On average the tissue loss in the isolated LAS treatment was 53 ± 30% (n = 8). The amount of tissue ablation was always similar in both fragments originating from the same coral colony. The tissue loss hampered the measurements of photosynthetic activity. During the first measurements at the beginning of the measurement phase all coral tissues were still intact and maximum quantum yield in the corals was 0.73 ± 0.01 (Fig 3). Due to the high loss of coral tissue, resulting in a separation of coral host and symbiotic zooxanthellae, no measurements of photosynthetic yield were obtained at the end of the experiment.

### Combined effects

Even though neither diesel nor temperature alone had a significant effect on oxygen fluxes, significant effects were found in the combined treatment. A significant interaction between diesel and temperature was detected (p = 0.0174 for net photosynthesis and p = 0.0029 for dark respiration, see Table 4). The Tukey test validated this effect only for the dark respiration (p = 0.0107, net photosynthesis p>0.07, see S1 Table in the supporting information). The net photosynthesis in the combined diesel and high temperature treatment was 0.0083 ± 0.0024 mgO_2_ h^-1^ cm^-2^ and dark respiration was 0.0227 ± 0.0028 mgO_2_ h^-1^ cm^-2^, thus higher than in the treatment with increased temperature alone (Fig 2). But there was no significant difference between the combined and the control treatment. There was no difference in gross photosynthesis (0.0144 ± 0.0051 mgO_2_ h^-1^ cm^-2^), P:R ratio (0.61 ± 0.15) or maximum quantum yield (0.72 ± 0.02).The combination of high temperature and LAS led to even higher tissue ablations than LAS in isolation (on average 92 ± 7%, with n = 8, in a range from 81 to 100%), thus results of maximum quantum yield were only obtained at the start of the measurement phase, when no ablations of coral tissue were yet visible. Maximum quantum yield in the combined LAS and high temperature treatment at this point was 0.63 ± 0.13 and thus significantly lower than the other treatments (p = 0.0265, Wilcox rank sum test).

## Discussion

### LAS and PAH in the environment

The analysis of ambient LAS and diesel concentrations around Gili Trawangan showed no measureable amounts of these pollutants in the water. This can be explained by the fact that the island as a tourism hotspot is kept clean and strong currents would quickly dilute any pollutants entering the water. A contrasting situation occurs in more densely populated areas, such as the Thousand Island chain off the Indonesian capital Jakarta, where also the boat traffic is much higher, including large commercial vessels passing through the island chain. At the Thousand Islands, PAH values up to 0.23 mg L^-1^ and LAS of up to 0.9 mg L^-1^ were detected (Baum et al. in prep.). Other studies in the Indo- Pacific measured total PAH concentrations between 0.05 to 0.21 mg L^-1^ [31], and LAS concentrations in the Red Sea ranging from 0.001 to 0.03 mg L^-1^ [46]. During short periods, the concentrations can reach higher values close to pollutant sources [46,47]. One source of diesel and PAH is the regular evacuation of water from the bilge in ships. Total PAH concentrations next to a boat after discharging bilge water were still higher than the PAH concentrations in our experiments (0.9 mg L^-1^ 10 min after discharging), but would further dissolve after a longer time period. Similarly, LAS concentrations next to a boat after cleaning were still 1.3 mg L^-1^ (G. Baum et al., in prep). This shows that the pollutant concentrations used in this study are relevant in the environmental context. The decreasing PAH and LAS concentrations during the course of our experiments resemble the natural exposure conditions of corals in the reef, with initially higher concentrations that are decreasing over time. Fast degradation has been described before for LAS [29] and to some extent also for diesel [48]. Half-life time of LAS in seawater is ca. 6 days due to biodegradation and adsorption to suspended particles [49].

### General notes

The respiration and photosynthetic yield values measured in the experiments were comparable to those measured by other authors for different coral species [33,49–51]. The values given in our paper for gross photosynthesis are estimations for the actual rates of oxygen produced, calculated from the difference between dark respiration and net photosynthesis. This assumes that holobiont respiration during dark and light are the same, which is not always the case as mentioned by Lesser (2013) [9]. Therefore gross photosynthesis might be slightly underestimated by this method. Compared to other studies, the calculated P:R ratio of 0.63 was very low. Generally, a P:R ratio below 1.0 indicates lack of photosynthetically fixed carbon [52]. In our study, this is due to the low light intensities from the artificial aquarium light. The light intensities were only one third of light intensities measured in the reef, explaining why photosynthesis rates during the experiments were quite low. Still 60 μmol quanta m^-2^ s^-1^ were enough to result in photosynthesis in *P*. *verrucosa*. Although it is possible that the slight reduction in light intensities between the pre-treatment and measurement phase might have interacted with the treatments and caused minor changes in the physiological reaction, this was regarded to be negligible because the light change was only small and a reduction is assumed to create no further stress for the corals.

### Effects of diesel

Diesel exposure had no effect on the coral physiology. Other authors have reported negative effects of different sources of oil on corals, but the literature on the effects of diesel, other oil sources and PAH on corals is contradictory. While several studies report decreases in maximum quantum yield [53], tissue alterations [54] and damages to reproductive systems [55,56], there are also other studies where no effect on corals was found [47]. In a recent study on cold water corals, DeLeo et al. (2015) also did not detect effects of crude oil treatments alone on coral health and mentioned that initial negative effects could be mitigated, when the coral holobiont uses the hydrocarbon components as a nutrition source [57], as indicated before by Al-Dahash and Mahmoud (2013) [58]. In coral tissues, total PAH concentrations of 0.004–0.1 μg g^-1^ dry mass were determined, higher than in the surrounding sediments, indicating a bioaccumulation [59,60]. Most studies on threshold values of PAH were performed on fish and crustaceans. In general LC_50_ concentrations for various PAH types and exposure times range from 0.0005 to 32.5 mg L^-1^ in a wide range of marine taxa [61]. In sole morphological and physiological effects occurred at PAH concentrations in sediments ranging from 0.054 to 4 μg g^-1^ dry mass [62]. No threshold values for corals are described in the literature. PAH metabolic products can bind to the organisms DNA and cause severe carcinogenic damage, as well as lead to alterations of the immune system [24], change blood composition and tissue [25].

### Effects of LAS

The effect LAS had on the coral tissue was surprisingly severe. First tissue ablations started to become visible after only 48 h exposure to 0.9 mg L^-1^ and after 84 h large parts of the coral tissue were detached from the skeleton. Trials with higher concentrations of LAS (results not shown) even lead to tissue losses up to 100% within the first 24 h. These tissue ablations pose a severe threat to coral health, as the regeneration of tissue needs a lot of energy and time. Generally, marine species tend to be more sensitive to LAS than freshwater species. Surfactants reduce the water surface tension that aquatic organisms depend on [47], explaining the severe effect on coral tissue as the membrane properties are disrupted [57,63]. Anionic surfactants such as LAS can bind to proteins and peptides, resulting in an alteration of their structure and function [47]. Thereby, these pollutants can alter enzymatic activities within the metabolic pathways and affect the DNA [29,64]. Temara et al. (2001) determined average LC_50_ values for marine species to be 4.3 mg L^-1^ with no observed effect concentrations at 0.3 mg L^-1^, which is 2 mg L^-1^ lower than for freshwater species [65]. There are only very few studies on the effects of LAS on corals, most experimental and monitoring work focused rather on fish and other invertebrates than corals. Shafir et al. (2014) performed the first study on the toxicology of detergents on hard corals and found them to be much more sensitive than many other marine organisms. LC_50_ for *Pocillopora damicornis* was determined to be 2.2 mg L^-1^, for *Stylophora pistillata* even 1.0 mg L^-1^ in 24 h exposures. In their experiments genotype-specific mortality and adaptation in *P*. *damicornis* after several exposures was observed [46]. Such a genotype-specific response could explain the finding that in our experiments always both fragments from the same coral colony showed similar ablation rates.

### Effects of high temperature

High temperature on its own led to a significant increase in maximum quantum yield, but only in the beginning of the measurement period. This shows that the coral reacts immediately to the change in temperature, but this effect is mitigated over time. Exposure time has a strong influence on the effect of temperature. While longer exposure reduces the respiration rates in corals, short term exposures can increase both respiration and photosynthetic rates [41]. In *Montastrea annularis* elevated temperature reduced both photosynthesis and respiration after 6 h of exposure [33]. Caribbean corals showed minor decreasing effects on photochemical efficiency due to higher temperature in experiments lasting for 10 days [11]. Other studies detected negative effects on photosynthesis and respiration due to temperature stress as well [49], although this could not statistically be replicated in the current study, where only negative trends of high temperature were measured.

### Combined effects of diesel and high temperature

Even though no significant differences of either diesel or temperature alone could be detected, when diesel was combined with temperature, there was a significant interaction. Dark respiration increased compared to the temperature as a single stressor and was similar to the control. This combined effect can be explained by altered membrane properties due to the higher temperature, which affect fluidity and diffusion rates and thus chemical toxicity [66], In hard corals from the Florida Keys, Porter et al. (1999) found that increases in both temperature and salinity reduced respiration rates and if administered at the same time, the effect was mitigated during the first 36 h, but still led to death of all corals after longer exposure [33]. Synergistic effects on maximum quantum yield were also demonstrated before between high temperature and very high light stress in Japanese corals, where yield decreased even stronger at higher temperature stress [16]. Reynaud et al. (2003) found an antagonistic effect of temperature in combination with increased *p*CO_2_ on calcification and photosynthesis of *Stylophora pistillata*, but not on respiration [17].

### Combined effects of LAS and high temperature

LAS in combination with high temperature further resulted in a significant reduction of the photosynthetic yield at the beginning of the experiment. Although at later time points no photosynthetic yield could be determined due to the tissue loss, decreases in yield were already seen in corals without tissue loss, showing the importance of yield measurements as early warning indicators. Declines of photosynthetic yield in corals were also measured after cyanide exposure and sedimentation, where yield values down to 0.1 during stress were recorded [40,67].

### Consequences for coral reef management

This study gives further confirmation about the need for better local management in the face of global warming. Even if CO_2_ emissions will be reduced, global warming will continue in future [7]. For coral reef organisms in tropical areas, a seemingly minor increase of a few degrees can result in severe stress, particularly if other local stressors are present as shown in this study. While removing thermal stress is not achievable in the near future, coral reefs are able to recover when other stressors are removed, thus it will be critical to support resilience of reefs by changing human destructive activities [68]. Indonesia’s population is strongly reef-associated, and most of the country’s coastline is populated without effective sewage treatments in place [2]. However, every family in each of the villages along the coast is using diesel and soap. Although at the moment no obvious effects of diesel or surfactants are visible on coral reefs, this could potentially become a problem in near future, taking growing populations and climate change into account, and expressing the need for effective local management [2,69]. Understanding the effect of different stressors and their combinations on key organisms can strengthen the decision support needed for coral reef management [8]. One way to reduce the outflow of pollutants from human settlements is the implementation of sewage treatments. Experiments on the effectiveness of sewage treatments have shown that from influent waters with LAS concentrations up to 6.7 mg L^-1^, only max. 0.005 mg L^-1^ remained in the effluent water [70]. In areas like the Netherlands, where 83% of all waste water is treated, LAS concentrations of only 1–9 μg L^-1^ were measured in nearshore estuaries [65]. Further controls on the direct discharge of diesel at least for commercial ships are necessary. At the same time, effective reef management can be achieved by communication of findings, education, training and outreach of populations living close to reefs to make them aware of the risks and the positive outcome when protecting the reefs [2].

### Conclusions

The physiology of *Pocillopora verrucosa* was influenced by the three stressors temperature, diesel and LAS in different ways. In combination with higher temperatures that are expected due to climate change, more severe responses of the coral to the pollutants were found. This confirms other studies, which have predicted a negative effect of global warming on organisms’ sensitivity to chemical stressors [14]. In addition, our study highlights the importance of measuring several response parameters, as different stressors can result in diverse responses. Further experiments with different concentrations of pollutants and the determination of threshold values, especially of LAS, would increase our knowledge on their effects on coral physiology. This study emphasizes the need to reduce local stressors such as LAS and diesel in order to support the survival of coral reefs facing of global warming.

## Supporting Information

S1 Table Results from post-hoc tests for diesel and temperature treatments. (DOCX)
