# Supplementary material for: Physiological Response of the Hard Coral Pocillopora verrucosa from Lombok, Indonesia, to Two Common Pollutants in Combination with High Temperature
Source: PLoS One. 2015 Nov 10;10(11):e0142744. doi: 10.1371/journal.pone.0142744 (PMC4640544; doi:10.1371/journal.pone.0142744)
Supplement: S1 Table — (DOCX) [file pone.0142744.s001.docx]

|  |  | diff | lwr | upr | | p adj |
| --- | --- | --- | --- | --- | --- | --- |
| **PAM Beginning of measurement period** | |  |  |  | |  |
| $Temperature | high-control | 0.0275 | 7.49E-03 | 0.047514 | | 0.0112 * |
| $Pollutant | none-diesel | 0.0000 | -2.00E-02 | | 0.020014 | 1.0000 |
| $`Temperature:Pollutant` | control:diesel-control:none | 0.0025 | -3.61E-02 | | 0.041067 | 0.9973 |
|  | high:diesel-control:none | 0.0275 | -1.11E-02 | | 0.066067 | 0.2026 |
|  | high:none-control:none | 0.0300 | -8.57E-03 | | 0.068567 | 0.1503 |
|  | high:diesel-control:diesel | 0.0250 | -1.36E-02 | | 0.063567 | 0.2689 |
|  | high:none-control:diesel | 0.0275 | -1.11E-02 | | 0.066067 | 0.2026 |
|  | high:none-high:diesel | 0.0025 | -3.61E-02 | | 0.041067 | 0.9973 |
| **PAM End of measurement period** | |  |  | |  |  |
| $Temperature | high-control | 0.0025 | -0.015702 | | 0.020702 | 0.7699 |
| $Pollutant | none-diesel | 0.0050 | -1.32E-02 | | 0.023202 | 0.5606 |
| $`Temperature:Pollutant` | control:none-high:diesel | 0.0025 | -3.26E-02 | | 0.037576 | 0.9965 |
|  | control:diesel-high:diesel | 0.0025 | -3.26E-02 | | 0.037576 | 0.9965 |
|  | high:none-high:diesel | 0.0100 | -2.51E-02 | | 0.045076 | 0.8315 |
|  | control:diesel-control:none | 0.0000 | -3.51E-02 | | 0.035076 | 1.0000 |
|  | high:none-control:none | 0.0075 | -2.76E-02 | | 0.042576 | 0.9188 |
|  | high:none-control:diesel | 0.0075 | -2.76E-02 | | 0.042576 | 0.9188 |
| **Light respiration** |  |  |  | |  |  |
| $Temperature | control-high | 0.0015 | -1.61E-03 | | 0.004597 | 0.3149 |
| $Pollutant | diesel-none | 0.0016 | -1.51E-03 | | 0.004697 | 0.2850 |
| $`Temperature:Pollutant` | control:diesel-high:none | 0.0031 | -2.89E-03 | | 0.009067 | 0.4494 |
|  | control:none-high:none | 0.0054 | -5.59E-04 | | 0.011399 | 0.0803 |
|  | high:diesel-high:none | 0.0055 | -4.59E-04 | | 0.011499 | 0.0739 |
|  | control:none-control:diesel | 0.0023 | -3.65E-03 | | 0.008312 | 0.6627 |
|  | high:diesel-control:diesel | 0.0024 | -3.55E-03 | | 0.008412 | 0.6338 |
|  | high:diesel-control:none | 0.0001 | -5.88E-03 | | 0.006079 | 1.0000 |
| **Dark respiration** |  |  |  | |  |  |
| $Temperature | high-control | 0.0000 | -4.19E-03 | | 0.004281 | 0.9824 |
| $Pollutant | diesel-none | 0.0033 | -8.99E-04 | | 0.007576 | 0.1117 |
| $`Temperature:Pollutant` | control:diesel-high:none | 0.0033 | -4.87E-03 | | 0.011461 | 0.6395 |
|  | control:none-high:none | 0.0072 | -9.49E-04 | | 0.015384 | 0.0899 |
|  | high:diesel-high:none | 0.0106 | 2.43E-03 | | 0.018766 | 0.0107 * |
|  | control:none-control:diesel | 0.0039 | -4.24E-03 | | 0.012089 | 0.5079 |
|  | high:diesel-control:diesel | 0.0073 | -8.61E-04 | | 0.015471 | 0.0853 |
|  | high:diesel-control:none | 0.0034 | -4.78E-03 | | 0.011549 | 0.6210 |

Tukey HSD test performed for the control, high temperature and diesel treatments. Temperature was either “control” (28 °C) or “high” (31 °C) and pollutant was either “none” or “diesel” (490 mL of 0.5 % WAF). Asterisks indicate significant effects (p<0.05).
